# Biomechanical evaluation of percutaneous cement discoplasty combined with percutaneous vertebroplasty: a finite element analysis

**DOI:** 10.3389/fbioe.2025.1606709

**Published:** 2025-07-08

**Authors:** Xu Zhou, Shenghua Qin, Chunhai Huang, Feiwen Li, Jin Liu, Ti Wu, Mingzheng Zhang

**Affiliations:** ^1^ Department of Orthopaedics, The First Affiliated Hospital of Jishou University, Jishou University, Jishou, China; ^2^ School of Medicine, Jishou University, Jishou, China

**Keywords:** spinal degeneration, biomechanics, finite element analysis, percutaneous cement discoplasty, percutaneous vertebroplasty

## Abstract

**Introduction:**

Since the introduction of percutaneous cement discoplasty (PCD), numerous studies have confirmed its clinical efficacy in elderly patients. However, PCD is also associated with risks such as bone cement leakage and vertebral fractures. The purpose of this study was to present a biomechanical evaluation of two modified versions of PCD performed in combination with percutaneous vertebroplasty (PVP).

**Methods:**

Data from a CT scan of a healthy male’s lumbosacral region were used to establish finite element (FE) models of nonsurgical treatment, PCD, L4/5PCD + L4L5PVP (modified technique 1, where the bone cement in the L4/5 disc space does not connect with the L4 and L5 vertebrae) and PCIF (modified technique 2, where the bone cement in the L4/5 disc space connects with the L4 and L5 vertebrae). A compressive of preload 150 N and a moment of 10 N·m were applied to recreate flexion, extension, lateral bending, and axial rotation. The range of motion (ROM) of L3/4 and L4/5, maximum stress on the L3 inferior endplate, L4 inferior endplate and L5 superior endplate, stress on the annulus fibrosus of L4/5, and displacement of the bone cement were evaluated.

**Results:**

Both modified techniques outperformed the simple PCD technique in reducing stress on the endplate, stress on the annulus fibrosus, and displacement of the bone cement. The L4/5PCD + L4L5PVP technique was more advantageous in terms of reducing the incidence of postoperative complications. The addition of the PVP technique significantly enhanced spinal stability by increasing support to adjacent vertebrae, thereby reducing the risk of postoperative endplate fractures and bone cement leakage.

**Conclusion:**

Modified PCD combined with PVP may be a safer and more effective option for treating degenerative disc diseases, providing important references for clinical treatment.

## 1 Introduction

With the aging of society, the incidence of spinal degenerative diseases continues to increase. Degenerative disc diseases cause spinal instability which leads to severe back pain and radiating leg pain, significantly diminishing patients’ quality of life and increasing the economic burden on families and society (GBD 2015 Disease and Injury Incidence and Prevalence Collaborators, 2016). Conservative treatments have limited efficacy, and traditional open surgeries are often associated with high rates of complications, such as wound infections (Mihailidis et al., 2017). There is an urgent need for new and safe minimally invasive surgical treatments. In 2015, Varga et al. (Varga et al., 2015) were first to report the effectiveness and safety of percutaneous cement discoplasty (PCD) for patients with intractable mechanical back pain and vacuum disc phenomena. This technique involves injecting bone cement into the nucleus of the degenerated disc to maintain the disc height and achieve indirect foraminal decompression, showing promising clinical results (Kiss et al., 2019; Camino et al., 2020). However, because bone cement cannot effectively fuse with the endplate cartilage and has a high elastic modulus, there are risks of postoperative complications such as endplate fractures, implant displacement, and adjacent vertebral fractures (Li et al., 2022; Grewal et al., 2024), which can exacerbate patients’ back and leg pain. These issues may stem from insufficient strength of adjacent vertebrae, lumbar instability, and displacement of bone cement within the disc space. Therefore, enhancing the strength of adjacent vertebrae, improving lumbar stability, and securing the bone cement within the disc space are crucial. Hence, we propose performing percutaneous vertebroplasty (PVP) of the adjacent vertebrae above and below the PCD. To achieve this, we propose two modified techniques: technique 1 (L4/5PCD + L4L5PVP, where the bone cement in the L4/5 disc space does not connect with the L4 and L5 vertebrae) or technique 2 (PCIF, where the bone cement in the L4/5 disc space connects with the L4 and L5 vertebrae) to promote fusion of the adjacent spinal motion segments (Xue et al., 2021). These modified techniques aim to reduce the risk of postoperative adjacent vertebral fractures and ensure stable anchoring of the bone cement within the disc space, minimizing the risks of displacement and PCD-related complications, providing a more effective and personalized surgical option for treating degenerative disc diseases and improving patients’ quality of life.

## 2 Methods

### 2.1 Establishment of the nonsurgical finite element model

**FIGURE 1:**
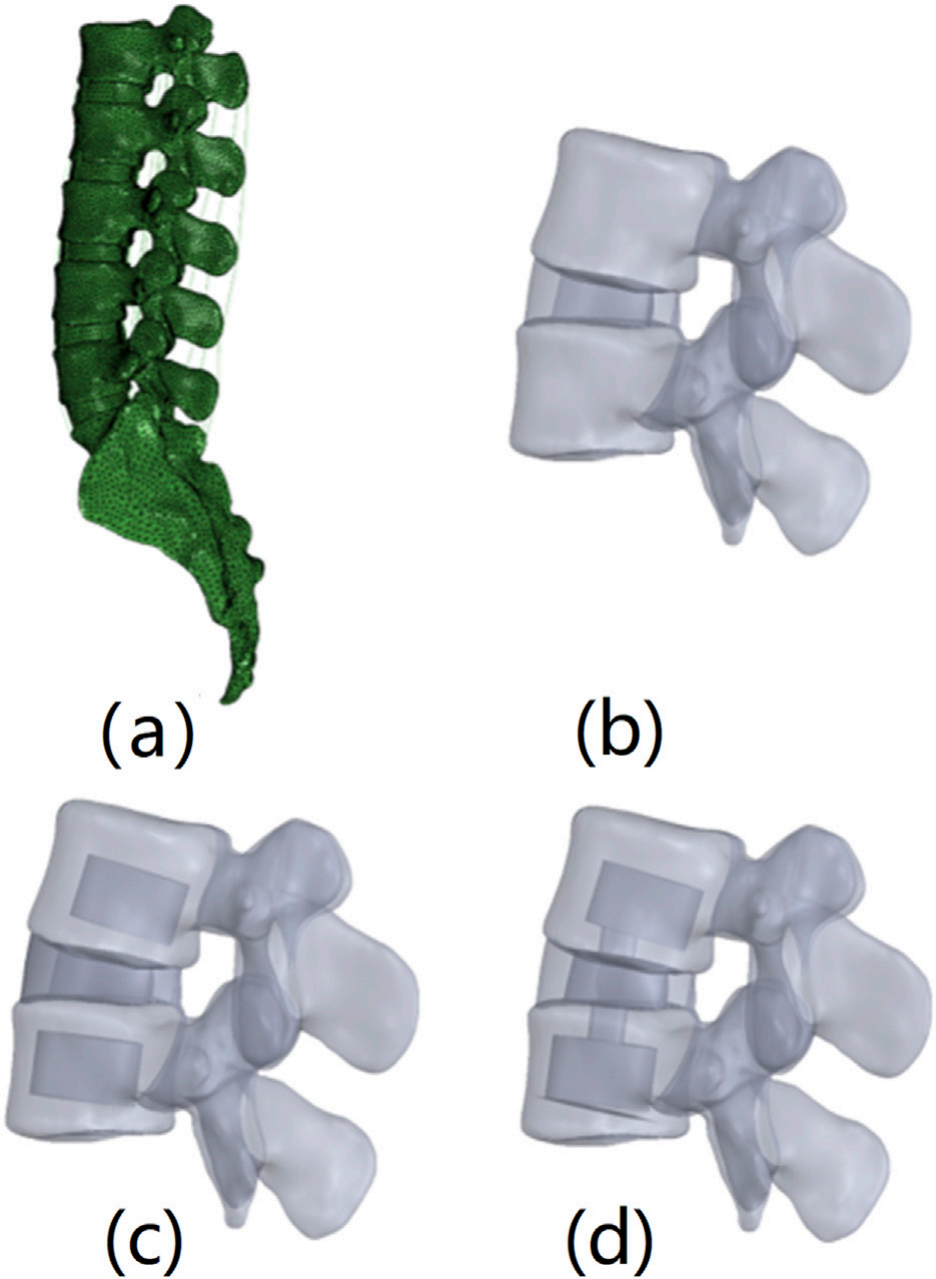
FE models of nonsurgical and surgical lumbar-sacral spines. **(A)** Nonsurgical model; **(B)** L4/5 PCD model (only the L4/5 segment is shown); **(C)** L4/5 PCD + L4L5 PVP model (only the L4/5 segment is shown); **(D)** PCIF model (only the L4/5 segment is shown).

CT of a healthy male’s lumbosacral region was performed with a slice thickness of 0.63 mm and a resolution of 0.39 mm × 0.39 mm, resulting in 776 DICOM format images. The lumbar CT images were imported into Mimics 10.01 (Materialise, Belgium) for vertebral segmentation and 3D geometric model reconstruction and exported in STL format. The STL-formatted vertebral models were imported into Geomagics Studio 12.0 (Raindrop Geomagics, Inc., U.S.) for smoothing and surface generation, producing NURBS surfaces, which were exported in IGS format. Since ligaments, such as the anterior longitudinal ligament (ALL), posterior longitudinal ligament (PLL), intertransverse ligament (TL), interspinous ligament (ISL), supraspinous ligament (SSL), capsular ligament (CL), and ligamentum flavum (LF) are not clearly visible in CT images, they were manually drawn as 3D lines in Mimics 10.01. Because the intervertebral disc is a soft tissue, it is difficult to segment from CT images, so it was drawn in CAD software Solidworks 2012 (Dassault Systèmes, France) in accordance with vertebral models and references, with the nucleus pulposus occupying 40% of the disc area (Chen et al., 2001). All the geometric models were imported into ABAQUS 6.11 (Simulia, Inc., USA) to establish the finite element model. The vertebrae consisted of the vertebral body and posterior elements, with the vertebral body further divided into cortical bone, cancellous bone, and the endplate. The cortical bone and endplate were modelled as 0.5 mm thick shell elements, whereas the other bony structures and discs were modelled as solid elements. Ligaments were modelled as two-node truss elements. The material properties were sourced from the literature (Tsuang et al., 2009; Fan et al., 2010; Ambati et al., 2015; Ellingson et al., 2015; Guo et al., 2025) and are detailed in Table 1. The full lumbar finite element model included the L1–L5 vertebrae, sacrum, and connecting discs and ligaments (Figure 1a).

**TABLE 1:** Material properties of the lumbar finite element model.

Tissue type	Young’s modulus (MPa)	Poisson’s ratio	Cross-sectional area (mm^2^)	Abaqus section type	Abaqus element type
Bony structure					
Cancellous bone	100	0.2	-	Solid	C3D4
Cortical bone	12,000	0.3	-	Shell	S3
Posterior elements	3500	0.25	-	Solid	C3D4
Endplates	3000	0.25	-	Shell	S3
Sacrum and coccyx	3500	0.25		Solid	C3D4
Ligaments					
ALL	15	—	40	Truss	T3D2
PLL	10	—	20	Truss	T3D2
SSL	8	—	30	Truss	T3D2
LF	15	—	40	Truss	T3D2
ISL	10	—	40	Truss	T3D2
TL	10	—	1.8	Truss	T3D2
CL	7.5	—	30	Truss	T3D2
Intervertebral disc					
Nuclei pulposi	1	0.499	—	Solid	C3D4
Annuli fibrosi	4.2	0.45	—	Solid	C3D4
Bone cement	3000	0.3	—	Solid	C3D4

### 2.2 Establishment of the surgical finite element model

Three surgical models were established: the L4/5 PCD model (Figure 1b), the modified technique 1 (L4/5PCD + L4L5PVP) model (Figure 1c), and the modified technique 2 (PCIF) model (Figure 1d). For the PCD model, the nucleus pulposus in the L4-L5 segment was replaced with bone cement. L4/5PCD + L4L5PVP: In addition to PCD, PVP was performed on the vertebrae above and below the affected disc. PCIF: After windows were created in the endplates above and below the affected disc, PCD was performed, followed by PVP on the adjacent vertebrae. Bone cement was modelled as a solid element with an elastic modulus of 3000 MPa and a Poisson’s ratio of 0.3.

### 2.3 Boundary conditions, model loading, and FE analysis

The facet joints were modelled as sliding friction with a coefficient of 0.1 (Zhong et al., 2009). The contacts between the endplate and vertebral body, the endplate and nucleus pulposus, the endplate and annulus fibrosus, and the bone cement and endplate/cancellous bone were set as tied. The sacrum was fully fixed to simulate the fixation method used in the biomechanical testing of cadaveric samples. A geometric reference point was selected at the centre of the L1 superior surface and coupled to the L1 surface for load application. A compressive preload of 150 N and a moment of 10 N·m were applied to recreate flexion, extension, lateral bending, and axial rotation. The data were collected and analysed post calculation. The evaluation indicators included (1) the ROM of L3/4 and L4/5; (2) the maximum stress on the L3 inferior endplate; (3) the maximum stress on the L4 inferior endplate; (4) the maximum stress on the L5 superior endplate; (5) the maximum displacement of bone cement in the disc space; and (6) the maximum von Mises stress on the L4/5 annulus fibrosus.

## 3 Results

### 3.1 FE mesh sensitivity analysis

**FIGURE 2:**
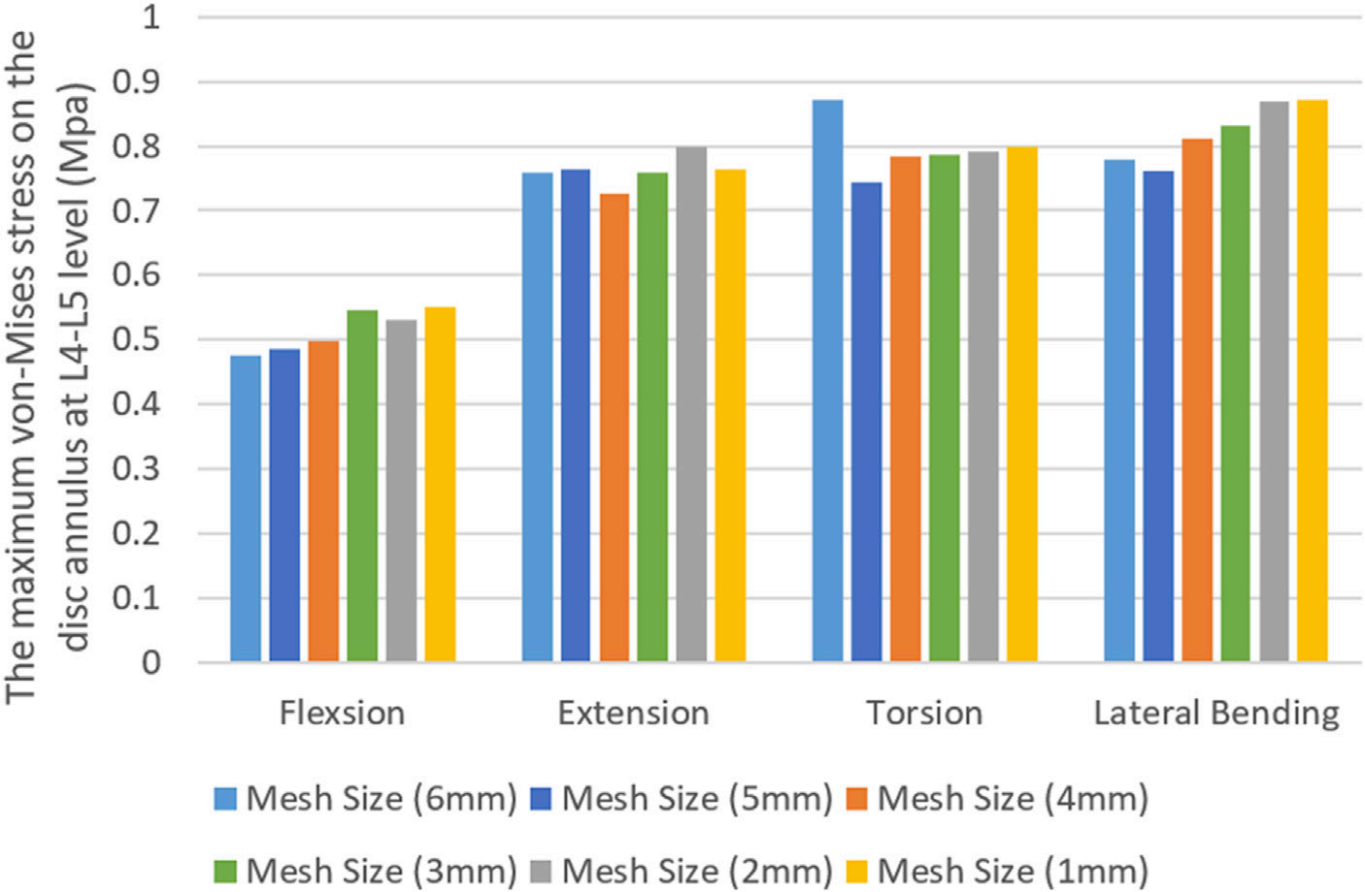
FE mesh sensitivity analysis.

The maximum von Mises stress on the L4/5 annulus fibrosus was used to perform a mesh sensitivity analysis of the model. The vertebrae of the nonsurgical model were meshed with mesh sizes ranging from 6 mm to 1 mm, and the results were shown in Figure 2. In general, reducing the mesh size improved result accuracy due to enhanced numerical convergence. Under all four loading conditions, the discrepancy in results between the 2-mm and 1-mm meshes was within 5%. After balancing computational efficiency and precision, we ultimately adopted a 2-mm mesh size for the vertebral meshing. During the meshing process, mesh quality control parameters specified in Table 2 were applied.

**TABLE 2:** Quality control parameters of the FE meshes.

Quality control parameters	Quadrilateral element	Triangular element	Tetrahedral element
Aspect ratio	<5.0	<5.0	<5.0
Jacobian ratio	>0.7	>0.7	>0.7
Internal angles	45°–135°	20°–120°	20°–120°

### 3.2 Model validation

**FIGURE 3:**
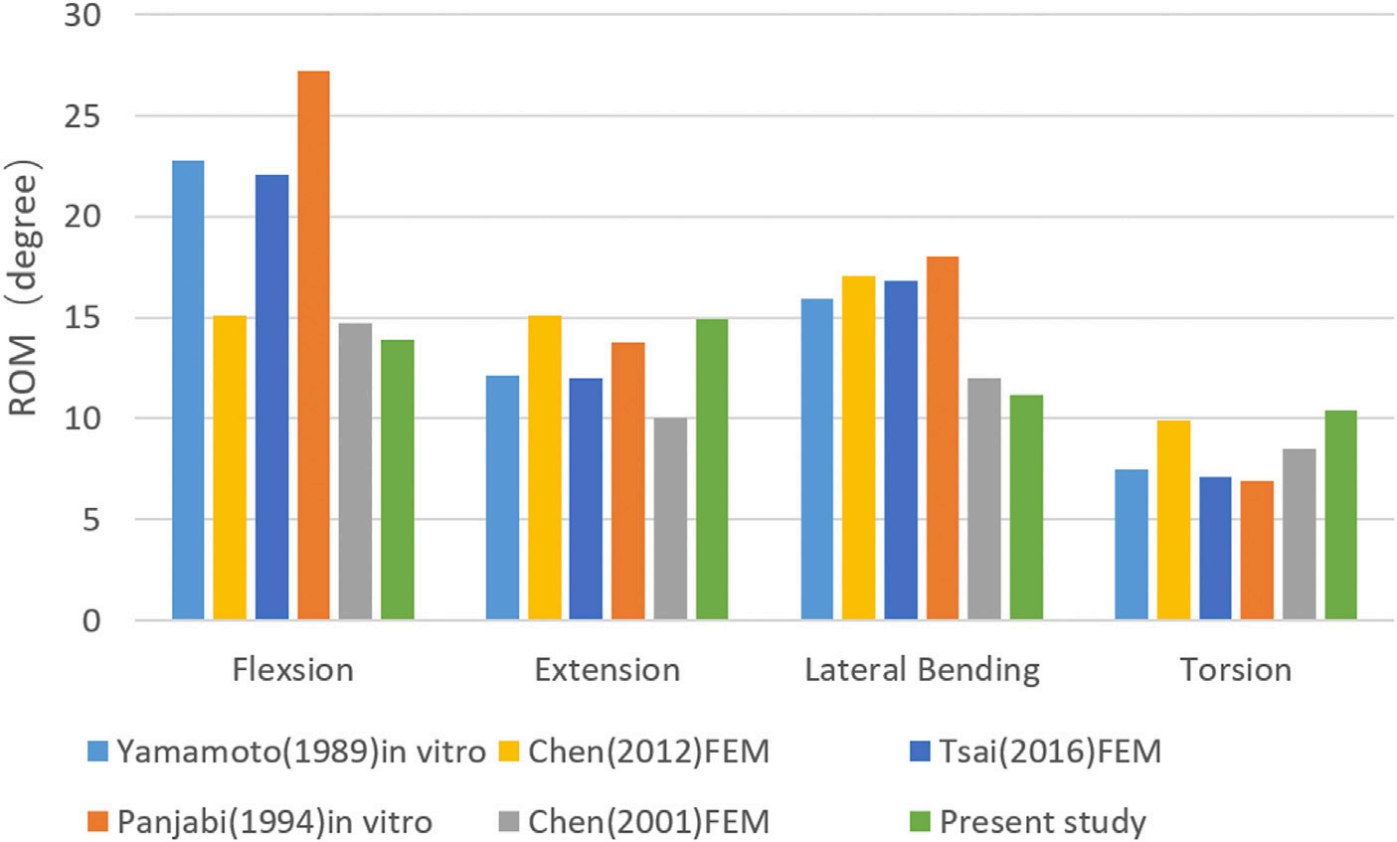
Model validation.

To validate the model, the ROM of the L1-L5 vertebrae in the nonsurgical model was compared with that in previous *in vitro* experiments (Yamamoto et al., 1989; Panjabi et al., 1994) and computational models (Chen et al., 2001; Chen et al., 2012; Tsai et al., 2016). As shown in Figure 3, the ROM of the L1–L5 vertebrae in the nonsurgical model was 13.74°, 14.70°, 11.17°, and 10.74° during flexion, extension, lateral bending, and rotation, respectively, showing good consistency with previous results.

### 3.3 The ROM of the L3/4 and L4/5 segments

**FIGURE 4:**
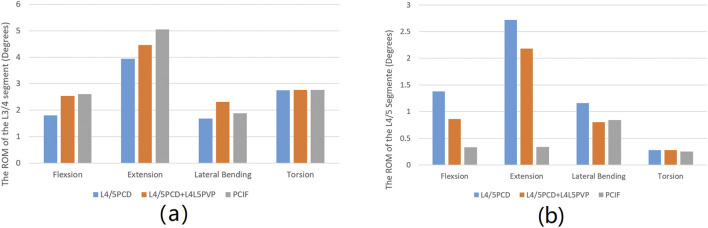
Range of motion. **(A)** Range of motion of the L3/4 segment; **(B)** Range of Motion of the L4/5 segment.

In the L4/5PCD model, the L3/4 segment had the lowest ROM in all loading conditions, with values of 1.80° during flexion, 3.94° during extension, 1.68° during lateral bending, and 2.75° during rotation. The ROM of the segments in all loading conditions was similar in the L4/5PCD + L4L5PVP and PCIF models (Figure 4a; Table 3). The ROM of the L4/5 segment in all loading conditions was greater in the L4/5PCD model than in the L4/5PCD + L4L5PVP and PCIF models, with values of 1.38° during flexion, 2.72° during extension, 1.16° during lateral bending, and 0.28° during rotation (Figure 4b; Table 3).

**TABLE 3:** Analysis results comparison table.

FE models	Loading conditions	ROM (°)		Maximum von mises stress (MPa)				Bone cement displacement (mm)
		L3/4 segment	L4/5 segment	L3 inferior endplate	L4 inferior endplate	L5 superior endplate	L4/5 annulus fibrosus	
L4/5 PCD	Flexion	1.80	1.38	30.90	25.61	20.50	0.24	2.71
	Extension	3.94	2.72	54.82	53.66	46.96	0.65	4.64
	Lateral bending	1.68	1.16	27.51	26.59	27.86	0.33	1.12
	Torsion	2.75	0.28	16.17	20.10	23.13	0.13	4.53
L4/5 PCD + L4L5 PVP	Flexion	2.54	0.86	29.41	12.35	14.18	0.14	1.81
	Extension	4.46	2.18	47.76	24.25	29.32	0.33	1.82
	Lateral bending	2.31	0.80	26.65	15.31	21.82	0.22	0.88
	Torsion	2.77	0.28	15.79	15.53	18.62	0.10	0.86
PCIF	Flexion	2.60	0.33	30.60	21.90	20.76	0.21	2.54
	Extension	5.05	0.34	51.67	36.65	44.16	0.51	4.70
	Lateral bending	1.89	0.84	27.21	23.23	17.66	0.27	1.01
	Torsion	2.76	0.25	16.48	17.09	19.42	0.11	1.60

### 3.4 Maximum von mises stress on the endplates

**FIGURE 5:**
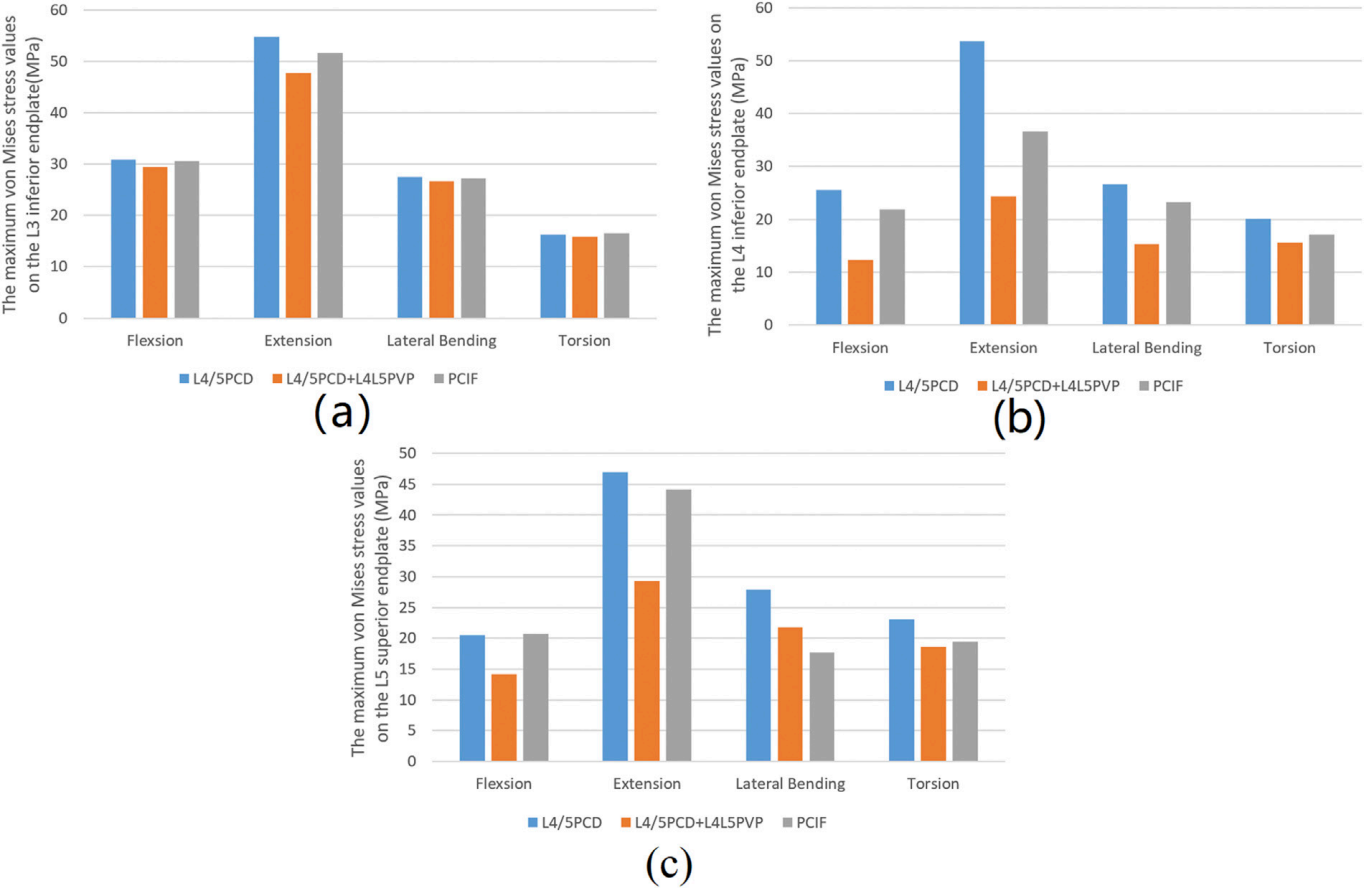
Maximum von Mises stress values. **(A)** Maximum von Mises stress values on the inferior endplate of L3; **(B)** Maximum von Mises stress values on the inferior endplate of L4; **(C)** Maximum von Mises stress values on the superior endplate of L5.

The maximum von Mises stress on the L3 and L4 inferior endplates and the L5 superior endplate in all four loading conditions was lowest in the L4/5PCD + L4L5PVP model; however, the von Mises stress on the L5 superior endplate during lateral bending was greater in the L4/5PCD + L4L5PVP model than in the PCIF model. The differences between the L4/5PCD + L4L5PVP and PCIF models were minimal (Figures 5; Table 3).

### 3.5 Maximum von mises stress on the L4/5 annulus fibrosus

**FIGURE 6:**
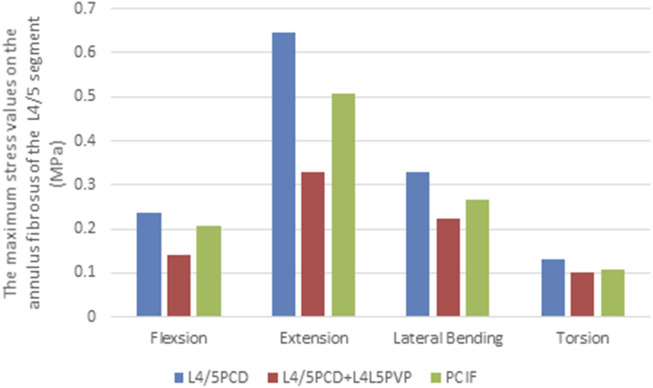
Maximum stress values on the annulus fibrosus of the L4/5 segment.

The maximum stress on the L4/5 annulus fibrosus in all four loading conditions was lower in both models than in the PCD model. L4/5PCD + L4L5PVP resulted in the lowest stress, with 0.1399 MPa during flexion, 0.6461 MPa during extension, 0.3297 MPa during lateral bending, and 0.1003 MPa during rotation (Figure 6; Table 3).

### 3.6 Bone cement displacement

**FIGURE 7:**
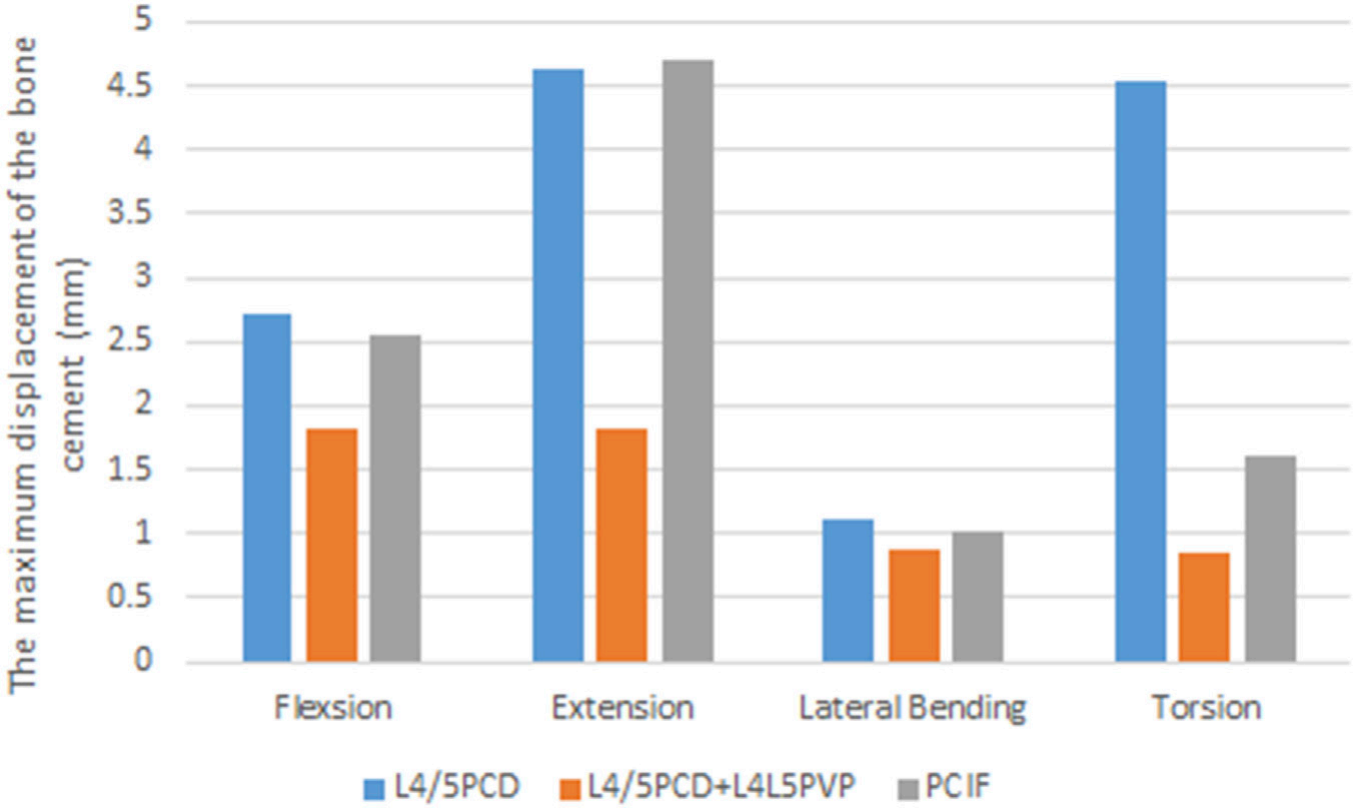
The maximum displacement of the bone cement.

The maximum displacement of bone cement in the disc space during flexion, extension, and lateral bending was similar in all four loading conditions in the L4/5PCD and PCIF models. However, PCIF significantly reduced displacement during rotation. L4/5PCD + L4L5PVP resulted in the smallest displacement (Figure 7; Table 3).

## 4 Discussion

Since the introduction of PCD, numerous studies have explored its effectiveness (Willhuber et al., 2021; Jia et al., 2022; Li et al., 2022; Techens et al., 2022; Zhang et al., 2024) and subsequently confirmed its clinical efficacy in elderly patients with vacuum disc phenomena and lumbar instability. However, PCD is associated with risks such as bone cement leakage and vertebral fractures (Kiss et al., 2019; Camino-Willhuber et al., 2021; Yamada et al., 2021). After PCD, the bone cement acts as an independent disc spacer but cannot fully fuse with the endplate cartilage. The annulus fibrosus, which is often incomplete or weak, may be further damaged during surgery, increasing the risk of cement leakage and displacement, similar to cage displacement in lumbar fusion surgery. Instability and osteoporosis progression after PCD are significant factors contributing to cement displacement (Kimura et al., 2012; Lee et al., 2018; Park et al., 2019). PCD does not provide sufficient stability or achieve solid interbody fusion, making cement displacement a critical concern. In this study, L4/5PCD + L4L5PVP resulted in the smallest degree of cement displacement, indicating its efficacy for securing the cement. The addition of the PVP technique enhances support to adjacent vertebrae, effectively securing the cement and reducing the risk of implant displacement.

Techens et al. (2020) reported the stress concentration on the endplates adjacent to the PCD-treated discs in an *in vitro* biomechanical study, which was consistent with the stress concentration calculated in a sheep model examined by Ghandour et al. (2022). To address the risk of adjacent endplate fractures, we compared the maximum stress on the L3 inferior endplate, L4 inferior endplate, and L5 superior endplate between PCD and the two modified techniques. Compared with PCD, both L4/5PCD + L4L5PVP and PCIF reduced the maximum stress on the L3 inferior endplate, indicating that reinforcement provided by PVP does not increase the risk of adjacent vertebral fractures. This advantage stems from the ability of PVP to distribute stress, reduce the burden on the endplates and lower the risk of postoperative fractures.

The ROM of the L3/4 segment was the lowest in the L4/5PCD model, indicating the strongest restriction on spinal motion, likely due to the high elastic modulus of the disc after cement injection. The ROM of the L4/5 segment in the L4/5PCD model was higher, indicating limited improvement in stability. In contrast, L4/5PCD + L4L5PVP and PCIF significantly reduced ROM, with PCIF providing the strongest stability as it fuses adjacent vertebrae and the cement in the affected disc space, reducing the risk of adjacent vertebral fractures and cement displacement. Li et al. (2022) also used finite element analysis to study PCD, confirming its impact on lumbar ROM and strength. The fusion of bone cement with endplates offers more biomechanical advantages, reducing the risk of cement subsidence and improving segmental stability.

The analysis of stress on the annulus fibrosus revealed that, compared with PCD, both L4/5PCD + L4L5PVP and PCIF reduced the maximum stress on the L4‒5 annulus fibrosus in all motion directions, with L4/5PCD + L4L5PVP showing the best results. This finding indicates that the addition of PVP led to effective stress distribution, reducing the burden on the annulus fibrosus and lowering the risk of postoperative annulus rupture.

Although the biomechanical effects of PCD and the two modified techniques were compared via finite element analysis, several limitations should be noted. For example, single subject CT images were used, thus limiting the generalizability of the results. A larger sample of patients of different sexes, ages, and weights should be selected for future studies to improve the applicability of the findings. In terms of model construction, simplified material parameters were used: the ligaments were modelled as two-node truss elements, while annulus fibrosi and endplates were modelled as isotropic linear elastic materials. The above simplifications may not accurately capture the complex kinematic characteristics of the spine. Muscular contributions were not incorporated in the simulation, and the study exclusively focused on static analyses without dynamic simulations. Subsequent studies ought to overcome these limitations by developing more refined models, such as utilizing three-dimensional solid ligaments with nonlinear properties to simulate ligaments and incorporating dynamic loading simulations to capture time-dependent behaviours. Furthermore, in future research, clinical follow-up data should be included to further validate the accuracy and reliability of the finite element analysis results.

## 5 Conclusion

In conclusion, both L4/5PCD + L4L5PVP and PCIF outperformed PCD in reducing the maximum stress on the endplate, maximum stress on the annulus fibrosus, and maximum bone cement displacement. Compared with PCIF, L4/5PCD + L4L5PVP was associated with fewer postoperative complications. The use of these techniques together enhance spinal stability by increasing support to adjacent vertebrae, which reduces the risk of postoperative complications. For patients with degenerative disc diseases, especially elderly patients, the combination of these techniques may be a safer and more effective treatment option, providing important references for clinical treatment.

## Data Availability

The raw data supporting the conclusion of this article will be made available by the authors, without undue reservation.

## Abbreviations

ALL, anterior longitudinal ligament; CL, capsular ligament; FE, finite element; ISL, interspinous ligament; LF, ligamentum flavum; PCD, percutaneous cement discoplasty; PCIF, percutaneous cement interbody fusion; PLL, posterior longitudinal ligament; PVP, percutaneous vertebroplasty; ROM, range of motion; TL, intertransverse ligament; SSL, supraspinous ligament.
